# Ketofol versus fentofol as induction agents for endotracheal intubation in emergency room

**DOI:** 10.1186/1865-1380-8-S1-P6

**Published:** 2015-04-22

**Authors:** Vijay Shankar Manickam, Prakash Mohanasundaram

**Affiliations:** 1 PSG institute of Medical Sciences & Research Hospital, Coimbatore, Tamil Nadu, India; 2 PSG IMSR & Hospital, Coimbatore, Tamil Nadu, India

## Objectives

To compare the effectiveness of Propofol plus Ketamine and Propofol plus Fentanyl as Procedural Sedation and Analgesia for Endotracheal Intubation in Emergency Department.

## Methods

The study was a randomized, double blinded study conducted between April 2014 and September 2014 in our Tertiary care Research Institute and Hospital. All Patients presenting to Emergency Department in need of Endotracheal Intubation were included in the study. The drugs used as Induction agents for Endotracheal Intubation were randomly numbered in Pre-filled syringes every day. Propofol was given in a dose 1.5 mg/kg (100 mg) for all patients in study population. Ketamine at 1 mg /kg (Group A) and Fentanyl at 1 µg/kg (Group B) were numbered and kept in 10 ml Prefilled syringes, either one of which was administered along with Propofol.

The primary outcomes were based on the Sedation level using the Ramsay Sedation Scale (RSS) and adverse respiratory events based on Quebec Criteria. Secondary outcomes were based on Hypotension, Cormack Lehane Grading, gag reflex, successful attempts at Intubation, oral and tracheobroncheal secretions, sedation time, procedure time, recovery time, recovery agitation and Optic nerve sheath diameter.

Patients with Cardio-respiratory arrest, age less than 18 years, head injury, glaucoma, hypotension, evidence of raised ICP and failure to give consent were excluded from the study.

## Results

A total of 286 patients were eligible for the study, of which 104 were included. 54 patients were randomized to Group A (Propofol plus Ketamine) and 50 patients were randomized to Group B (Propofol plus Fentanyl). RSS of ≥ 5 was considered adequate. 45 (83.33%) patients in Group A and 39 (78%) patients in Group B had adequate sedation. Overall Incidence of Adverse respiratory events was less in Group A with 9 (9.25%) patients compared with Group B with 16 (32%) patients. Secondary outcome criteria had minimal statistically significant difference, especially reduced incidence of hypotension in Group A compared to Group B.

## Conclusion

Ketofol provided better analgesia & sedation, lesser adverse respiratory events and reduced incidence of post-induction hypotension compared to Fentofol, as an induction agent for endotracheal Intubation among patients in our Emergency Department.

